# Research on LEACH Protocol Based on Dynamic Clustering and Routing Optimization

**DOI:** 10.3390/s26010199

**Published:** 2025-12-27

**Authors:** Tongtong Wang, Xingye Qu, Huiqing Cui

**Affiliations:** College of Engineering, Yanbian University, Yanji 133002, China; 2023050054@ybu.edu.cn (T.W.); 2024050060@ybu.edu.cn (X.Q.)

**Keywords:** WSN, LEACH protocol, cluster head election, multi-hop transmission, route optimization

## Abstract

The limited and often irreplaceable battery energy of Wireless Sensor Network (WSN) nodes, which are typically deployed in harsh environments, poses a critical challenge. Excessive energy consumption can lead to node failure and consequent data loss, making energy efficiency a central research focus. To address the limitations of the LEACH protocol in cluster head (CH) election and transmission modes, this paper proposes an optimized approach. First, sensor nodes are clustered using a Self-Organizing Map (SOM) neural network. Subsequently, the CH election function incorporates the node’s residual energy, distance to the base station, and neighbor node density. Finally, the data transmission stage employs a hybrid method combining Fibonacci sequences and a bee algorithm for routing optimization. The simulation results demonstrate that the proposed protocol outperforms benchmarks in terms of the node death round, network lifetime, and data throughput across different base station locations, offering a valuable technical solution for routing optimization in medium- and large-scale WSNs.

## 1. Introduction

Communication technologies for the Internet of Things (IoT) primarily include wireless sensor networks (WSNs), wireless communication, mobile communication, and satellite remote sensing [1]. A WSN consists of numerous sensor nodes that monitor various environmental information—such as temperature, humidity, sound, and radionuclides—and transmit the collected data to a Base Station (BS). The BS then sends this data to user terminals via wired or wireless means for further processing [2]. However, sensor nodes are constrained by limited energy and bandwidth, and their batteries are often impractical to charge or replace [3]. This makes energy efficiency the foremost challenge in WSNs. Layered routing protocols are a key technology in this field, and designing an efficient one is vital for improving node energy efficiency, reducing consumption, and extending network lifetime [4].

The LEACH (Low Energy Adaptive Clustering Hierarchy) protocol is a classic adaptive clustering protocol and the first to perform data fusion within a hierarchical framework [5]. To balance network energy consumption, LEACH randomly selects cluster head (CH) nodes in periodic rounds, with each round comprising a cluster establishment phase and a stable data transmission phase [6]. This approach ensures that every node has an equal probability of serving as the CH, promoting relatively uniform energy consumption across the network and helping to extend its overall lifetime [7].

### 1.1. Improvements in the Cluster Establishment Phase

However, the LEACH protocol is not without its shortcomings. A significant disadvantage lies in the randomness of its CH election function, which can lead to unbalanced energy consumption across network nodes and consequently shortening the overall network lifecycle. To address this, Xiong et al. [8] effectively balanced energy consumption during CH election by introducing a triple optimization strategy involving high-order nodes, dynamic energy perception thresholds, and centralized computation at the BS, thereby mitigating the issue of concentrated node death. Nevertheless, this improved mechanism relies heavily on a pre-deployed heterogeneous network environment, limiting its applicability in common homogeneous networks. Similarly, Li et al. [9] proposed a multi-factor CH election mechanism that incorporates energy, distance, and round number, permitting the re-election of high-energy nodes. Shi et al. [10] also reconstructed the election threshold by introducing multiple operators and dynamically adjusted weights via a fuzzy analytic hierarchy process, constructing a multi-factor cost function for the clustering stage. While these studies exemplify a sophisticated approach, they depend on complex threshold functions and introduce considerably higher computational overhead, presenting a fundamental challenge for resource-constrained sensor nodes. Senturk [11] integrated a three-layer feedforward neural network into the CH election process using node state parameters as input to achieve rapid election, which significantly enhanced decision efficiency and suitability for large-scale real-time networks. However, the offline training process for such a model demands substantial computational resources, and the resulting static model lacks an intrinsic mechanism to adapt to long-term network dynamics, such as persistent energy depletion. Thiagarajan et al. [12] employed a particle swarm optimization (PSO) algorithm to dynamically select CHs based on energy and distance, aiming to equalize energy consumption within clusters. A limitation here is that the effectiveness of PSO is highly contingent on the careful tuning of its parameters (e.g., inertia weight, acceleration coefficients), and its performance may degrade in dynamic network environments, thus limiting its robustness and practicality. M. Revanesh et al. [13] utilized an improved Levenberg–Marquardt neural network during cluster formation and introduced a sub-CH mechanism to distribute the load on the primary CH, effectively reducing intra-cluster energy consumption. Yet, the computational complexity of the neural network training leads to slow convergence in large-scale networks, and the signaling overhead required for dual-layer CH coordination can increase dramatically, potentially offsetting the energy savings gained from the structural improvements. Finally, Li et al. [14] determined the optimal CH ratio by integrating an energy model with calculations for node transmission and data fusion energy consumption. They refined the CH election threshold formula using residual energy and distance factors and, during cluster formation, considered cluster scale density to prevent significant disparities in node count among clusters.

### 1.2. Improvements to Transmission Modes and Paths

A further limitation of the LEACH protocol lies in its data transmission mode, which is inefficient for long-distance communication. To address this, Mao et al. [15] proposed a forwarding satisfaction function for the data transmission stage, which rapidly screens relay nodes based on their energy, distance, and angle to form an adaptive multi-hop chain, thereby alleviating the bottleneck of single-hop transmission. However, their node screening mechanism, which is reliant on fixed thresholds, lacks adaptability to dynamic network topologies and may lead to relay node overload. Wu et al. [16] introduced a cuckoo search algorithm for the inter-cluster stage, constructing a satisfaction function based on energy and distance to achieve efficient multi-hop routing by rapidly iterating towards optimal relay nodes for extreme-value clusters. Founder [17] employed an improved ant colony algorithm in inter-cluster transmission to dynamically plan multi-hop paths, updating pheromones according to path length and node energy to balance energy consumption. N. Meenakshi et al. [18] proposed an eagle fragment optimization system based on passive clustering, which optimizes CH selection from multiple dimensions and constructs an optimal path to the BS using a wing ant-fly optimization algorithm to enhance energy efficiency and throughput. A drawback of this approach is that its path construction algorithm is prone to local optima, limiting its performance in large-scale, dynamic networks. Separately, Unalido Ntabeni et al. [19] applied mean field game theory to dynamically adjust the transmission power of CHs during data transmission, although this work did not address the inherent randomness in the CH election process itself.

Although existing studies on the LEACH protocol have demonstrated improvements in CH quality by integrating metrics such as residual energy, node distribution density, and distance to the BS, a significant limitation persists. These strategies often overlook the dynamic operational characteristics of the network, including perpetual node energy decay, real-time topological shifts, and imbalanced load distribution across different areas, which jointly hinder the extension of network longevity. In the realm of routing optimization, the application of intelligent algorithms for path selection, while effective, shows limited adaptability when confronted with diverse network scales and application scenarios. Prevailing solutions are typically confined to localized refinements, focusing solely on isolated parameters like distance or energy and thus fall short of providing a holistic framework that synergizes the clustering mechanism with the routing strategy. Consequently, the co-operative relationship between cluster formation and multi-hop routing necessitates deeper exploration.

Based on our previous research, the LEACH-PM protocol [20] significantly enhanced the performance of the classical LEACH protocol by introducing a uniform clustering mechanism and an election function that comprehensively considers both node residual energy and distance to the BS. This protocol demonstrates excellent energy consumption balance in small to medium-scale static network environments. However, its static cluster architecture based on fixed partitions struggles to adapt to dynamically changing topologies in large-scale networks, resulting in noticeable bottlenecks in scalability and energy efficiency.

Furthermore, many prevalent intelligent clustering approaches, such as K-means, primarily focus on optimizing the geometric distribution of nodes based on distance. This single-factor optimization often results in spatially balanced clusters that are nonetheless blind to energy status.

Based on the above overview, existing improvements mainly follow three approaches: those focusing solely on CH election, those concentrating only on routing-path optimization, and those that combine both. Methodologically, they range from heuristic algorithms to neural networks. The clustering and routing phases typically operate based on static snapshots of network conditions. This is the key reason why they struggle to adapt to large-scale networks with dynamically changing topologies.

To overcome this fundamental limitation and address other deficiencies in existing research, this paper proposes the LEACH-DR protocol. This innovative protocol deeply integrates the dynamic clustering capability of self-organizing neural networks with the intelligent routing optimization mechanism of the Fibonacci-inspired bee algorithm, establishing an adaptive solution tailored for large-scale network environments. The main contributions of this paper are as follows:

1. It proposes a dynamic clustering mechanism based on a Self-Organizing Map (SOM), which uses node distance, neighbor density, and residual energy as unified inputs. This forms energy-aware and topologically stable cluster structures capable of continuous self-adaptation, overcoming the instability of distance-only methods such as K-means.
2. We designed a dual-stage CH election scheme, comprising a weighted fitness function for optimal primary CH selection and a similarity-based alternative CH pre-deployment mechanism. This ensures service continuity and reduces data loss through seamless failover.
3. We developed a Fibonacci-inspired bee algorithm for state-aware, multi-hop routing between CHs and the base station. The algorithm dynamically optimizes paths, effectively solving the inefficient long-distance single-hop transmission problem of the LEACH protocol.

The rest of the paper is structured as follows: Section 2 describes the relevant work, Section 3 describes the protocol methodology for this study, the simulation and experimental results are presented in Section 4, and finally, Section 5 summarizes the paper.

## 2. Related Work

**Figure 1:**
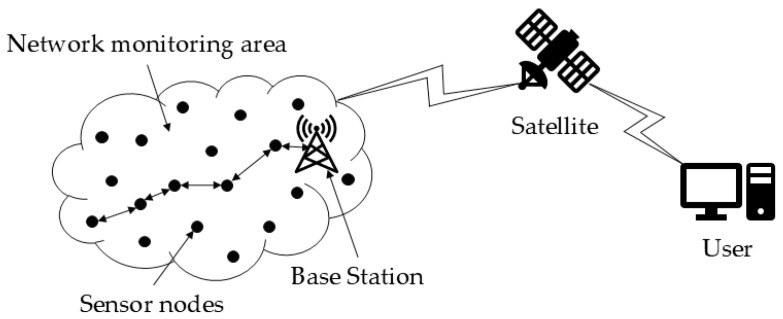
WSN architecture diagram.

A typical WSN architecture, as illustrated in Figure 1, comprises sensor nodes, CH nodes, and a BS. In this hierarchical model, sensor nodes initially monitor environmental data and transmit it to their respective CH. The CH then aggregates and fuses the data from all nodes within its cluster. Subsequently, this fused data is relayed to the BS, which finally forwards the consolidated information to the end-user.

The following assumptions are made for the network model: N sensor nodes are randomly distributed in a square monitoring area of 500 m × 500 m; Each sensor node has a unique node ID tag; All sensor nodes have the same and limited energy and cannot be replaced or stored; The results are calculated by the BS and broadcast.

### 2.1. LEACH Protocol

#### 2.1.1. Establishment Stage

In the LEACH protocol, CHs are elected primarily based on a threshold function, T(n). During each round, a node that has not served as a CH in the recent cycles generates a random number between 0 and 1. If this number is less than the current threshold T(n), the node becomes eligible to be a CH for the current round. Conversely, a node is disqualified and remains ineligible if it has been a CH in the preceding period or if its generated random number exceeds T(n). The specific formula for the CH election threshold is given in Equation (1).

(1) $$T\left(n\right)=\left\{\begin{array}{rr} \frac{p}{1-p\times \left[r\mathrm{mod}\left(\frac{1}{p}\right)\right]} & ,n\in G\\ 0 & ,n\notin G \end{array}\right.$$

where *p* is the probability that the node will be selected as the CH, *r* is the number of periods currently running, mod represents the remainder, and *G* is the collection of nodes that have not been selected as the CH. At the beginning, when the value of *r* is 0, the value of T(n) is *p* according to Equation (1), and all nodes in the network can participate in the election of CH nodes, and the probability of becoming a CH is *p*.

#### 2.1.2. Steady-State Stage

**Figure 2:**
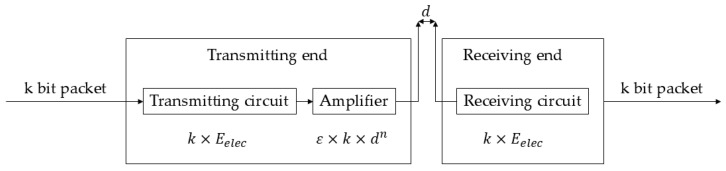
First-order radio model.

To calculate the energy consumption of the sensor nodes, the first-order radio model commonly used in WSNs is used, as shown in Figure 2.

The energy consumption of the sensor node to send *k*-bit data to the target at a distance d is calculated by Equation (2), and the value of d is determined by Equation (3):

(2) $$\begin{array}{cc} E_{Tx}\left(k,d\right) & =E_{Tx\text{-}elec}(k)+E_{Tx-amp}(k,d)\\ & =\left\{\begin{array}{c} kE_{elec}+k\varepsilon_{fs}d^{2},d<d_{0}\\ kE_{elec}+k\varepsilon_{mp}d^{4},d\geq d_{0} \end{array}\right. \end{array}$$

(3) $$d_{0}=\sqrt{\frac{\varepsilon_{fs}}{\varepsilon_{mp}}}$$

The energy consumption of receiving *k*-bit data is calculated by Equation (4):

(4) $$E_{Rx}\left(k\right)=E_{Rx-elec}\left(k\right)=E_{elec}k$$

The energy consumption of fused *k*-bit data is calculated by Equation (5).

(5) $$E_{DA}\left(k\right)=E_{DA}k$$

Among them, $E_{elec}$ represents the radio energy dissipation of sensor nodes in the transmitter or receiver circuit, $\varepsilon_{fs}$ is the power coefficient of the free-space model, $\varepsilon_{mp}$ is the power coefficient of the multipath fading model, $E_{DA}$ is the energy consumption coefficient for data fusion, and $d_{0}$ is the threshold distance for selecting the transmission model.

### 2.2. Self-Organizing Mapping Neural Networks

**Figure 3:**
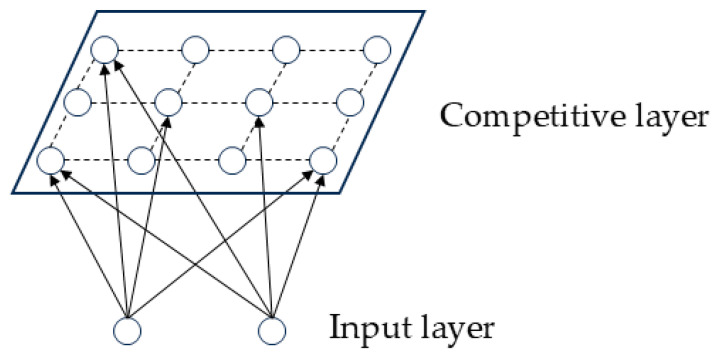
SOM neural network topology diagram.

Neural networks have achieved remarkable success in various fields and are now widely regarded as the most effective tools in artificial intelligence [21]. A self-organizing mapping (SOM) neural network is a self-organizing neural network for learning and training, consisting of an input layer and a competition layer. For new input samples, the network competition layer will produce different responses and correspond to different regions, and the formed response will act on neurons in the form of feedback to form a neural network topology map to obtain the output results [22]. The network topology is shown in Figure 3.

The SOM neural network possesses the inherent capability to autonomously determine the optimal number of clusters. This facilitates an even distribution of the workload across all sensor nodes, thereby minimizing the system’s overall energy consumption [23]. The primary objective of an SOM is to transform high-dimensional input data into a low-dimensional discrete map without relying on pre-existing labels, all while preserving the topological structure of the original data space. The underlying mechanism of the SOM operation is built upon three foundational principles: competition, cooperation, and adaptation [24].

The specific procedural steps for implementing the SOM neural network are as follows:

1. Initialize network parameters: Initialize the neural network by randomly initializing the weight vector of the output layer neuron nodes, ensuring that the dimensions match the input data *X*. For example, for the input vector $X=\left(x_{1},x_{2},...,x_{m}\right)$, the weight vector $W_{j}=\left(w_{j1},w_{j2},...,w_{jm}\right)$ for the neuron, where $w_{ji}$ represents the distance from the *j*-th neuron to the *i*-th component of the input vector *X*.
2. Randomly select sample inputs: Input data samples with size *n* × *m*, where *n* denotes the number of input vectors and *m* denotes the number of dimensions.
3. Obtain the best matching neuron: Calculate the Euclidean distance $d_{j\left(X\right)}$ between the *j*-th neuron and the input sample vector *X*, with the neuron having the minimum distance to the input sample identified as the required neuron *I*(*X*). The formula for the Euclidean distance is as shown in Equation (6):

(6) $$d_{j\left(X\right)}=\sum_{i=1}^{m}{\left(x_{i}-w_{ji}\right)}^{2}$$

**Figure 4:**
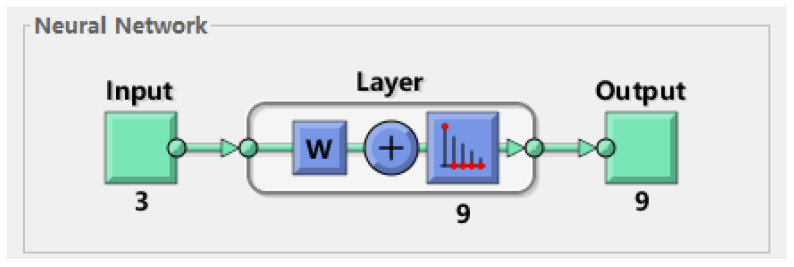
SOM network diagram.

4. Update the weight vector, topological neighborhood, and learning rate: After finding the best matching neuron *I*(*X*), update the weights of that neuron and its neighboring neurons according to Equation (7).
(7) $$\Delta w_{ji}=\eta \left(t\right)\cdot h_{I,j}\left(t\right)\cdot \left(x_{i}\right)-w_{ji}$$
  Here, η is the learning rate, and $h_{I,j}\left(t\right)$ is a time-varying Gaussian function used to obtain the topological neighborhood of the neuron.

5. Determine whether the SOM has terminated iterations: If the predetermined number of iterations is reached or the learning rate $\eta \left(t\right)$ falls below a certain threshold, the network is considered to have converged; otherwise, repeat steps 2 to 4.
  The self-organizing mapping structure diagram is shown in Figure 4.

### 2.3. Fibonacci Heuristic Bee Algorithm

The Fibonacci sequence was proposed by Leonardo Fibonacci and became popular in the 19th century [25]. The sequence starts with 0 and 1, and each number after that is added to the first two digits. The sequence is as follows: 0, 1, 1, 2, 3, 5, 8, etc. [26]. The algebraic definition can be expressed as $F_{0}=0$, $F_{1}=1$, and for any integer n≥2, there is

(8) $$F_{n}=F_{n-1}+F_{n-2}$$

This recurrence relation belongs to a second-order linear homogeneous difference equation with constant coefficients, and its characteristic equation.

(9) $$\gamma^{2}-\gamma -1=0$$

The two roots are $\varphi =\left(1+\sqrt{5}\right)/2$ (golden ratio) and $\psi =\left(1-\sqrt{5}\right)/2$, and the closed form formula of this sequence can be derived using the characteristic roots as follows:

(10) $$F_{n}=\frac{\left(\varphi^{n}-\psi^{n}\right)}{\sqrt{5}}$$

Since $\left|\varphi \right|$<1, for n sufficiently large $\varphi^{n}\to 0$, then $F_{n}\approx \varphi^{n}/\sqrt{5}$, leads to the limit relation $\lim_{n\to \infty }F_{n+1}/F_{n}=\varphi$, which reveals the connection between the Fibonacci sequence and the golden ratio.

The Bees Algorithm was originally developed for continuous problems with six user-determined parameters and is often referred to as the Basic Bees Algorithm (BBA) [27].

The Bees Algorithm is a swarm intelligence optimization technique inspired by the foraging behavior of honeybees. The algorithm simulates a colony comprising two primary roles: scout bees and forager bees (or employed bees). Scout bees are responsible for exploring the environment to discover nectar sources (potential solutions). Upon finding a source, a scout bee returns to the hive and recruits forager bees to exploit it. Scout bees are further categorized based on the quality (fitness) of the nectar source they discover. Those who find poor sources become random scouts and continue exploring randomly. Those who find promising sources become employed scouts and return to the hive to recruit foragers. Among these employed scouts, a subset of e bees that discovered the best fitness sources (where e < m) are designated as elite scouts. The remaining m-e bees are good scouts. Recruitment is proportional to source quality: an elite scout leads a larger number of foragers (nre), while a good scout leads a smaller contingent (nrb, where nrb < nre). Thus, the total number of foragers recruited is e × nre + (m − e) × nrb.

The pseudocode of the basic bee algorithm is shown in Algorithm 1:
**Algorithm 1:** Basic Bees Algorithm Pseudocode**Input:** n: number of scout bees, m: number of better scout bees, e: number of best scout bees, nrb: recruited bees for other better scout bees, nre: recruited bees for best scout bees, ngh: initial size of neighbourhood**1 Function** BBA(n, m, e, nrb, nre, ngh):**2    Initialise Population** with random solutions**3    Evaluate Fitness** of the population**4    While** stopping criterion not met **do****5**        Forming new population**6        Better Scout Bees** for neighbourhood search**7      Recruit Bees** for other better scout bees (more bees for the best scout bees) and evaluate fitness**8        Select Fittest Bees** from each patch**9        Assign Random Bees** to search randomly and evaluate their fitness**10    End****11 return** Best Bee

The optimal reconnaissance bee of the bee algorithm can search for the optimal relay node in the Fibonacci neighborhood, and the optimal reconnaissance bee can prevent falling into the local extreme value. The golden ratio decay law of the Fibonacci sequence provides an adaptive mechanism for the bee algorithm, which can effectively solve the problems of energy consumption hotspots and premature node death in the transmission stage of the LEACH protocol without adding additional communication overhead.

## 3. Problem Description and Formulation

To clearly define the scope and objectives of our research, this section formally describes the network model, key assumptions, and the optimization goals that the proposed LEACH-DR protocol aims to achieve.

### 3.1. Network Model and Assumptions

The proposed protocol was developed based on a static and homogeneous WSN with the following model and assumptions:

1. Network Deployment

A total of N sensor nodes are uniformly and randomly deployed in a square monitoring area of M × M (e.g., 500 m × 500 m).

2. Node Homogeneity

All sensor nodes are homogeneous. They are assigned the same initial energy level $E_{\mathrm{initial}}$ and possess identical capabilities in sensing, processing, and communication.

3. BS Configuration

A single stationary BS with fixed coordinates serves as the data sink. The BS operates with unrestricted power resources and sufficient computational capacity for protocol coordination.

4. Node Capabilities

Each node can determine its spatial position through integrated localization systems and can perform dynamic transmission power adjustment based on real-time distance calculations.

5. Energy Consumption Model

The first-order radio dissipation model (detailed in Section 2.1.2, Figure 2) governs all energy consumption during transmission, reception, and data aggregation operations.

6. Network Topology

The network topology remains quasi-static during operation, with topological changes occurring only through node failure from energy exhaustion.

### 3.2. Optimization Objectives

The LEACH-DR protocol is designed to maximize network longevity through synergistic optimization of clustering and routing mechanisms. The optimization objectives are formally specified using these quantifiable metrics:

1. Network Stability Extension

Maximize the operational rounds until First Node Demise (FND), ensuring sustained network coverage and data integrity during the critical initial operational phase.

2. Network Longevity Enhancement

Maximize the sustained network operation until a predefined node failure threshold is reached, typically measured as complete network energy exhaustion.

3. Data Delivery Maximization

Maximize the cumulative data packets successfully delivered to the BS throughout the complete network operational lifetime.

The proposed protocol achieves these objectives through the integrated implementation of dynamic cluster formation and adaptive multi-hop path selection, effectively addressing the fundamental limitations inherent in the conventional LEACH protocol architecture.

## 4. Clustering and Routing Optimization Protocols

### 4.1. SOM Configuration

To address the limitations of static clustering in dynamic WSNs, LEACH-DR introduces a SOM neural network to achieve dynamic and adaptive clustering. The configuration and rationale behind the SOM are detailed below.

1. Feature Selection and Normalization

At the beginning of each clustering round, three critical node attributes—residual energy, Euclidean distance to the BS, and the number of neighbor nodes—are collected and normalized to the [0, 1] interval using Equations (11)–(13).

(11) $$E_{factor}=\frac{E_{residual}}{E_{\max}}$$

(12) $$d_{factor}=\frac{d_{itoBS}}{d_{\max}}$$

(13) $$N_{factor}=\frac{N}{N_{\max}}$$

Here, $E_{residual}$ denotes the current residual energy of the node; $E_{\max}$ represents the maximum residual energy among all nodes in the network; $d_{itoBS}$ is the distance from the current node to the BS; $d_{\max}$ is the maximum distance from any node to the BS; N signifies the number of neighbor nodes for the current node; and $N_{\max}$ is the maximum number of neighbor nodes any node has. This process forms a comprehensive 3-dimensional feature vector that encapsulates the node’s energy status, spatial location, and local density, ensuring all factors contribute equally to the clustering.

2. SOM Structure and Training

The SOM’s competitive layer is configured as a fixed 3 × 3 grid (9 neurons), a design choice directly linked to the network scale of 200 nodes. This structure inherently constrains the number of clusters to 9, aiming for an average of 22–23 nodes per cluster, which strikes a balance between cluster management overhead and intra-cluster communication efficiency. The SOM undergoes 100 training iterations using the unsupervised learning process described in Section 2.2. This iterative process, governed by a Gaussian neighborhood function, ensures that nodes with similar energy, location, and density characteristics are mapped to the same or adjacent neurons.

3. Impact on Cluster Formation and Energy Efficiency

**Figure 5:**
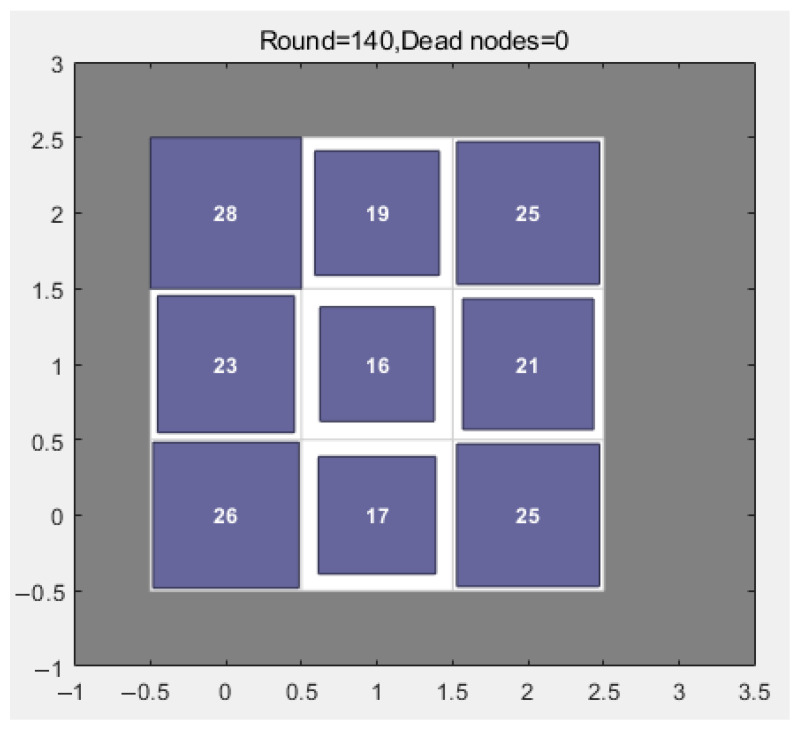
Distribution of the number of nodes in the cluster after SOM optimization.

This SOM configuration directly results in energy-balanced and topologically rational clusters. As evidenced in Figure 5, the final cluster sizes range from 16 to 28 nodes, with a max-min ratio of less than 2. This equilibrium is crucial for energy efficiency. It prevents the formation of overly large clusters that would burden a single CH with excessive data fusion and long-distance transmission, as well as overly small clusters that lead to CH overpopulation and increased inter-cluster routing overhead. By forming compact and balanced clusters, the SOM setup minimizes the average intra-cluster communication distance and effectively distributes the network load, thereby directly reducing the system’s overall energy consumption and extending the network lifetime.

After the SOM completes the clustering for a given round, how to scientifically select the cluster head nodes in each cluster becomes the key to affecting the energy consumption balance and operation life of the network.

### 4.2. Cluster Header Election Function

The traditional LEACH protocol uses a stochastic threshold mechanism, which is simple to implement, but does not consider factors such as the remaining energy and spatial location of the node, which can easily lead to communication bottlenecks. In order to optimize node energy consumption and extend the network life cycle, a fitness CH election strategy considering residual energy, spatial location, and neighbor density is introduced in the cluster. The core idea of this strategy is to give priority to nodes with high remaining energy that are located in key topological positions as CHs, which can control the communication overhead within the cluster and improve the data forwarding efficiency. The construction process of this fitness election strategy is described in detail below.

1. Residual energy factor

Define the energy factor of node i as Equation (14)

(14) $$E_{f}\left(i,t\right)=\frac{E_{re}\left(i,t\right)}{\bar{E_{re}\left(t\right)}}$$

among them, $E_{re}\left(i,t\right)$ is the remaining energy of node *i*, and $\bar{E_{re}\left(t\right)}$ represents the average remaining energy of the network nodes. If the value of $E_{f}\left(i,t\right)$ is greater than 1, it indicates that the energy of this node is higher than the network average, and its energy is sufficient to undertake long-distance transmission tasks, thereby reducing the risk of data loss due to insufficient energy. Conversely, if the value of $E_{f}\left(i,t\right)$ is less than 1, this node’s energy is relatively scarce and is not suitable for taking on the role of CH.

2. Distance factor

The communication distance between the cluster node and the BS directly determines the energy consumption, and in order to reduce the communication energy consumption of the long-distance node that becomes the CH node, the distance factor is defined as Equation (15)

(15) $$D_{f}\left(i,t\right)=\frac{d{\left(i,BS\right)}_{\max}-d\left(i,BS\right)}{d{\left(i,BS\right)}_{\max}}$$

here, $d\left(i,BS\right)$ is the Euclidean distance from node i to the BS, and $d{\left(i,BS\right)}_{\max}$ represents the maximum distance from any node in the network to the BS. From the formula analysis, it can be seen that the value range of $D_{f}\left(i,t\right)$ should be (0, 1]. The closer $D_{f}\left(i,t\right)$ is to 1, the nearer the node is to the BS, resulting in a higher success rate of data packet reception by the BS; conversely, the closer $D_{f}\left(i,t\right)$ is to 0, the more it indicates that the node is located at the edge of the network, which leads to significant energy consumption due to long-distance communication. By incorporating the distance factor into the CH election strategy, it can effectively suppress the additional energy consumption caused by electing distant nodes as CHs.

3. Number of neighbor nodes factor

If the CH node is located in a sparse area of nodes, the member nodes in the cluster need to increase the energy consumption to complete the communication, so the number of neighbor nodes factor is introduced.

(16) $$C_{f}\left(i,t\right)=\frac{\deg\left(i\right)}{N}$$

$\deg\left(i\right)$ is the number of neighboring nodes of node *i*, and N is the number of all nodes deployed in the network. The higher the $C_{f}\left(i,t\right)$ value, the higher the node is located in a topological high-density area, which can significantly shorten the average communication distance within the cluster and reduce the overall energy consumption. On the other hand, high-density areas can provide more CH options to build better communication links.

4. Build a fitness function

The comprehensive fitness function of node i is formed by linear weighting of the above three factors, as shown in Equation (17).

(17) $$F\left(i,t\right)=\frac{1}{z}\left[\alpha \cdot E_{f}\left(i,t\right)+\beta \cdot D_{f}\left(i,t\right)+\gamma \cdot C_{f}\left(i,t\right)\right]$$

where z=α+β+γ is a normalization constant, and the weighting coefficients were determined based on the optimal pairing identified through simulation experiments. A critical insight in constructing this function is the synergistic roles of the spatial factors $D_{f}\left(i,t\right)$ and $C_{f}\left(i,t\right)$ in defining the node’s position. The distance factor $D_{f}\left(i,t\right)$ quantifies the global spatial location relative to the BS, directly determining the immense energy cost of long-haul transmission. Conversely, the neighbor density factor $C_{f}\left(i,t\right)$ captures the local spatial context; a higher $C_{f}\left(i,t\right)$ value indicates a node resides in a dense region, which minimizes the average intra-cluster communication distance and load on the CH. The residual energy factor $E_{f}\left(i,t\right)$ serves as the fundamental constraint, ensuring the elected CH possesses sufficient endurance. A larger $N\left(i\right)$ value indicates a higher suitability for the node to become a CH. This multi-faceted strategy effectively suppresses the disturbances from energy estimation biases and the inherent randomness of stochastic elections, thereby ensuring stable and predictable CH selection, which is crucial for maintaining robust network links.

Alternative CH nodes can be set to fail when the main CH node runs out of energy or the channel fades suddenly, avoiding additional control overhead caused by reselecting the CH. After introducing the alternative CH, the constraints of real-time monitoring scenarios on data integrity are met. When setting the alternative CH, the number of common neighbors with the main CH node, the distance from the main CH, and the remaining energy of the node itself are mainly considered, and when the remaining energy of the alternative CH node is higher than that of the main CH node, it will replace the main CH node and become the new CH node. The following is a detailed explanation of the similarity function for constructing alternative CH nodes.

1. The number of common neighbors of nodes and CH nodes

Within the communication radius $R_{ci}$, the set of neighbor nodes that belong to both node *i* and the cluster head (CH) can be represented as:

(18) $$N_{overlap}\left(\left(i,CH\right),t\right)=N_{i}\left(t\right)\cap N_{CH}\left(t\right)$$

here, the cardinality $\left|N_{overlap}\left(\left(i,CH\right),t\right)\right|$ of the common neighbor set directly reflects the degree of topological overlap between the node and the CH. A larger value indicates that the node possesses a stronger local connectivity preservation capability within the cluster. Should the CH fail, this node can directly maintain and rapidly restore most of the original neighbor links, thereby minimizing the probability of network partitioning. The weighting factor for the number of neighbor nodes is defined as follows:

(19) $$C_{s}\left(\left(i,CH\right),t\right)=\frac{\left|N_{overlap}\left(\left(i,CH\right),t\right)\right|}{\left|N_{CH}\left(t\right)\right|}$$

where $\left|N_{CH}\left(t\right)\right|$ is the cardinality of the CH’s own neighbor set.

2. The distance between the node and the CH

The spatial separation between node *i* and the CH is calculated using the Minkowski Distance, as shown in Equation (20):

(20) $$D_{s}\left(i,CH\right)={\left|p_{i}-p_{CH}\right|}_{2}$$

where $p_{i}$ and $p_{CH}$ are the coordinate vectors of node *i* and the CH, respectively. Analysis indicates that the closer the distance, the easier it is for the node to maintain a transmission power similar to that of the CH, thereby ensuring minimal fluctuation in link budget after switching between the primary and alternate CHs. However, selecting the alternate CH based solely on the principle of minimizing distance may lead to energy hotspot problems. An energy hotspot refers to a situation where a few nodes in the network undertake excessive data forwarding or processing tasks, causing their energy consumption to be significantly higher than that of neighboring nodes, thereby leading to premature energy depletion.

Define its distance weighting factor as

(21) $$D_{s}\left(\left(i,CH\right),t\right)=\frac{1}{D_{s}\left(i,CH\right)}$$

3. The remaining energy of the node

To avoid the energy hotspot problem, the residual energy factor is defined as the ratio of the remaining energy of the node to the average remaining energy of the network, as shown in Equation (22)

(22) $$E_{s}\left(i,t\right)=\frac{E_{re}\left(i,t\right)}{\bar{E_{re}\left(t\right)}}$$

If the value of the remaining energy factor is greater than 1, it means that the node has the ability to undertake long-term tasks.

4. Construct a similarity function

Based on the factors mentioned above, a similarity function can be constructed as shown in Equation (23):

(23) $$S\left(\left(i,CH\right),t\right)=\eta_{1}\cdot C_{s}\left(\left(i,CH\right),t\right)+\eta_{2}\cdot D_{s}\left(\left(i,CH\right),t\right)+\eta_{3}\cdot E_{s}\left(i,t\right)$$

when $\eta_{1}+\eta_{2}+\eta_{3}=1$, the numerical distribution is determined by simulation experiments. The similarity function is composed of three factors: neighboring node factors, distance factors, and energy factors, which express the ability of the routing structure to inherit after replacing the main CH based on the topological overlap degree, the stability of the transmission link budget within the cluster based on distance constraints, and the sustainability of undertaking transmission tasks based on the relative remaining energy ratio. To enhance network robustness, an alternative CH is pre-selected within each cluster based on the highest similarity function value U(n). This node remains on standby, monitoring the main CH. The alternative CH is automatically activated under either of two conditions: (1) it detects that the main CH’s residual energy has dropped below its own or (2) it perceives a sudden and persistent failure in communication from the main CH (e.g., via channel fade). This pre-designation and energy-based trigger mechanism eliminates the need for a global re-clustering or a complex re-election process, thereby avoiding significant additional control overhead. The seamless transition ensures the persistence of the data link with minimal disruption. The constructed similarity function allows the system to maintain the persistence of the data link without incurring additional energy consumption after the current CH node fails.

### 4.3. Routing Optimization in the Data Transmission Phase

During the inter-cluster transmission phase of LEACH, the conventional single-hop direct transmission method causes remote CHs to suffer from excessive energy drain, rapidly leading to network lifetime imbalance. To address this critical issue, this paper introduces a novel approach that seamlessly integrates Fibonacci sequences into the bees algorithm for constructing optimized multi-hop routing paths. This hybrid strategy effectively mitigates the energy consumption imbalance inherent in single-hop paradigms while simultaneously reducing the network’s overall energy expenditure. Consequently, the proposed method demonstrates superior suitability for large-scale network deployments.

Specifically, the network formed by all CH nodes and the BS is abstracted as an Open Traveling Salesman Problem. This is distinct from a classic TSP as it does not require a closed loop; instead, it seeks an optimal path that starts from one CH, traverses all other CHs, and ends uniquely at the BS. The solution is thus the minimal-length, unclosed curve that connects the entire set of CHs to this single endpoint.

**Figure 6:**
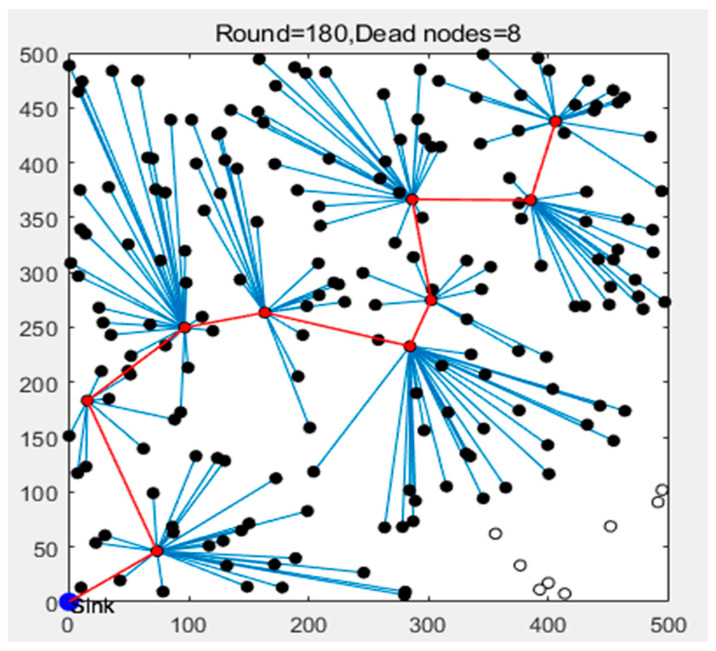
The optimized data transmission path.

In this paper, the Fibonacci-based bee algorithm is introduced to solve the optimal path length: n scout bees are set, among which, m only find feasible paths and are identified as the better scout bees, and the other n-m only continue to search feasible paths randomly within the network to maintain the global search ability. Among the m optimal paths, the first e paths are selected as the optimal path according to the fitness ranking, corresponding to e optimal scouts. Each optimal scout bee only recruits nre foragers to explore the path field in depth, and the other n-e better scouts only recruit nrb foragers (nrb < nre).The random step size of the scout bees and the neighborhood update of the foraging bees both adopt the Fibonacci golden ratio $\varphi =\left(1+\sqrt{5}\right)/2$, and the step size ratio decays exponentially with iteration. After iterative convergence, only one path with the lowest total cost is retained as the data transmission link, and all CH nodes forward the fused data to the BS along this link in turn. The transmission path in the network operation phase is shown in Figure 6.

In the routing optimization phase, the Fibonacci-inspired Bees Algorithm is employed to solve the optimal multi-hop path. The core innovation lies in using the golden ratio φ, derived from the Fibonacci sequence, to adaptively control the neighborhood search radius (ngh) for the scout and forager bees. Specifically, the neighborhood size decays exponentially according to the rule $ngh\left(t\right)=ngh\left(t-1\right)*\left(\varphi -1\right)$, where φ−1≈0.618. This provides a natural and efficient decay mechanism, balancing global exploration and local exploitation. A larger neighborhood in early iterations promotes exploration, while a finer, shrinking neighborhood in later iterations facilitates precise convergence. This dynamic adjustment prevents the algorithm from stagnating in local optima and leads to more energy-balanced paths by thoroughly searching the solution space before refining the best candidates.

Based on the above, the working steps of the LEACH-DR protocol can be described as follows:

1. In the network initialization stage, the BS broadcasts to the whole network to wake up all nodes, and after receiving the broadcast, the node judges its position by the strength of the received signal, and then encapsulates its own ID, energy, and position information into a frame, and transmits it back to the BS, completing the initialization stage and preparing to enter the cluster establishment stage;
2. The nodes normalize the remaining energy, the distance from the BS, and the number of neighboring nodes to obtain a three-dimensional eigenvector. After collecting all the feature vectors, the BS runs a SOM network with an output layer of 3 × 3 grids, and after 100 iterations of training, all nodes of the network are divided into 9 clusters with balanced energy and topology. After the training is completed, the BS transmits the cluster division to each node, and after the node confirms the cluster group to which it belongs, it enters the CH election stage.
3. The BS application Equation (17) calculates the fitness function value for each cluster, and the node with the highest fitness value is determined as the CH node, and the BS broadcasts the information. At the same time, the BS calculates the similarity function value for other nodes in the cluster according to Equation (23), and the node with the highest function value is determined as the alternative CH node, and the other nodes are used as member nodes. When the remaining energy of the alternative CH node is higher than that of the CH node, the alternative CH node becomes the main CH node.
4. The CH node counts the number of members in the cluster, generates a TDMA schedule, and sends it to the members in the cluster.
5. After the CH node completes the data collection in the cluster, the data is fused and processed, and the shortest and optimal multi-hop transmission path is calculated by using the improved bee algorithm inspired by Fibonacci, and the fused data is forwarded to the BS, hop by hop along the path.
6. If the number of surviving nodes is greater than 0, repeat steps (2)~(5) until all nodes die and the network runs end.

**Figure 7:**
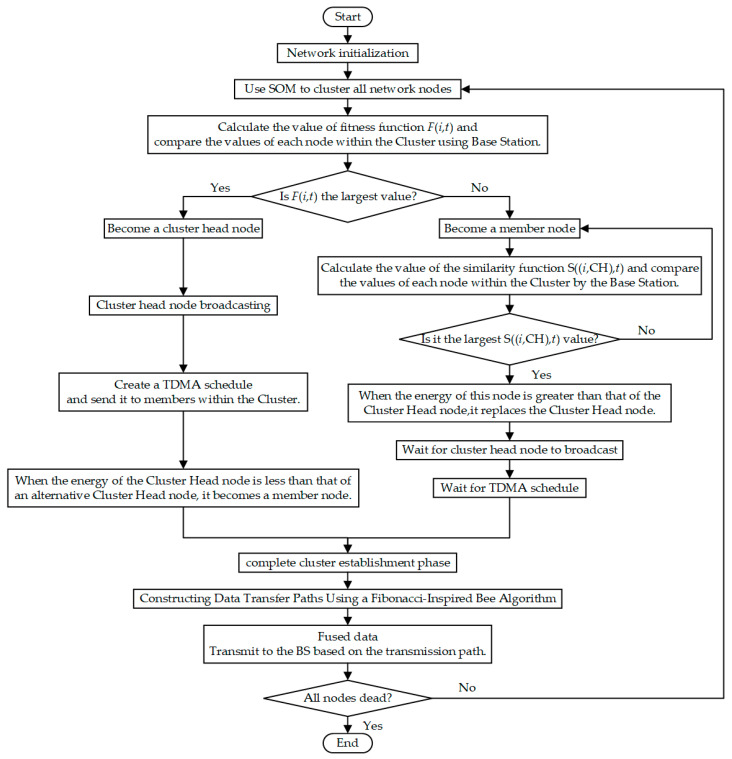
LEACH-DR protocol flow chart.

The flow chart of the LEACH-DR protocol is shown in Figure 7:

### 4.4. Analysis of Computational and Communication Overheads

The LEACH-DR protocol performs SOM training and bees algorithm optimization centrally at the Base Station (BS), without imposing additional computational burden on sensor nodes. SOM training is executed once per round with a complexity of $O\left(k\cdot n\cdot m\right)$, where *k* is the number of iterations (set to 100 in this paper), *n* is the number of nodes, and *m* is the number of output neurons (set to 9). The bees algorithm for path planning also runs on the BS with controllable iterations and fast convergence.

The additional communication overhead mainly includes: BS broadcasting cluster assignments per round and nodes reporting status information (energy, location, neighbor count). The control message overhead scales linearly with the number of nodes N, and its energy consumption is far lower than the energy saved through dynamic clustering and intelligent routing.

In medium- to large-scale WSNs, the BS typically possesses sufficient computational and energy resources, making the overhead of LEACH-DR acceptable and scalable.

## 5. Simulation Results and Analysis

**Figure 8:**
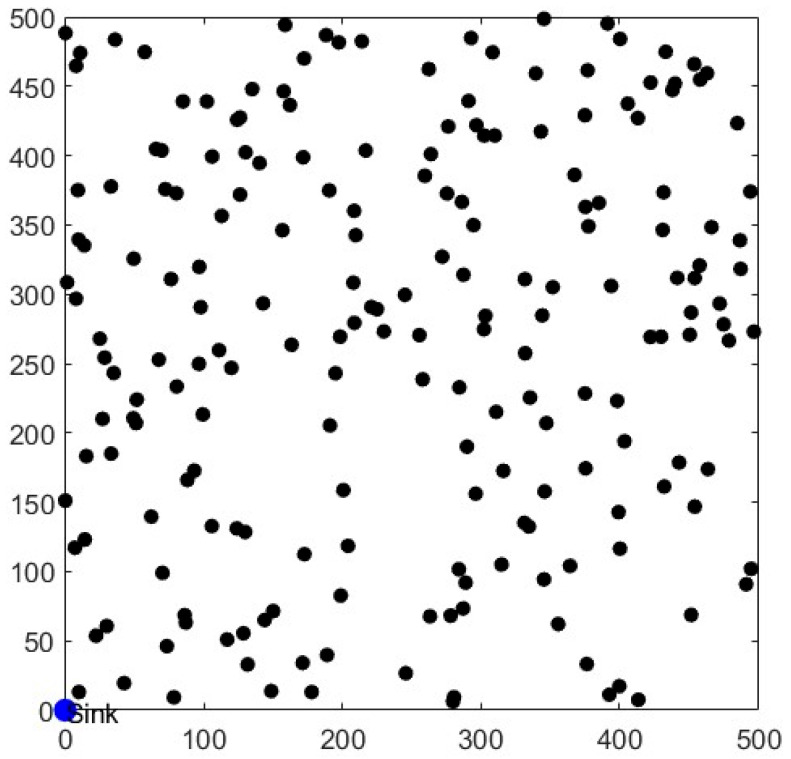
Network node distribution map.

In this study, 200 nodes were randomly sown in a 500 m × 500 m square monitoring area, and the deployed node locations are shown in Figure 8. All presented results are based on the average of 50 independent random deployments to ensure statistical reliability. The LEACH-DR protocol was simulated and analyzed using MATLAB R2022a simulation software, and compared with LEACH [5], LEACH-PM [20], and LEACH-EI [14]; the simulation parameters are shown in Table 1.

1. Network residual energy analysis

**Figure 9:**
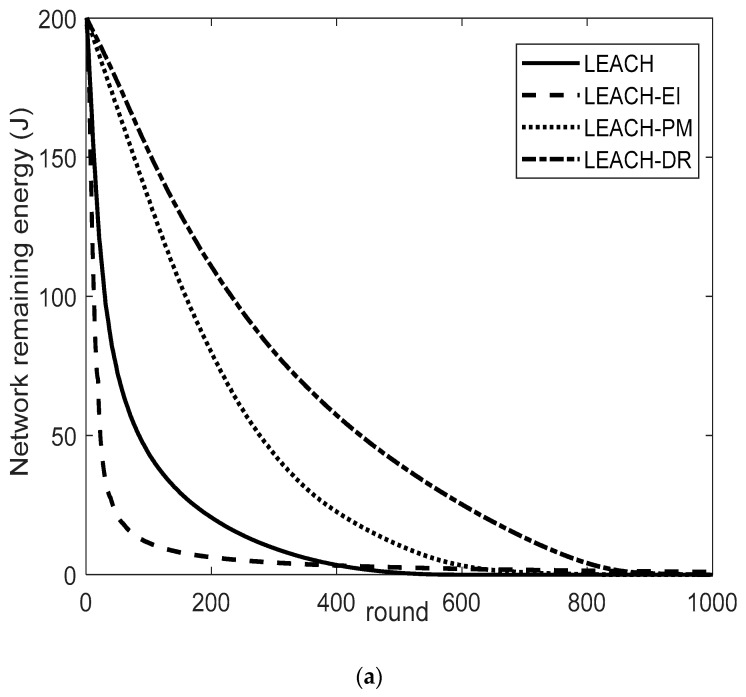

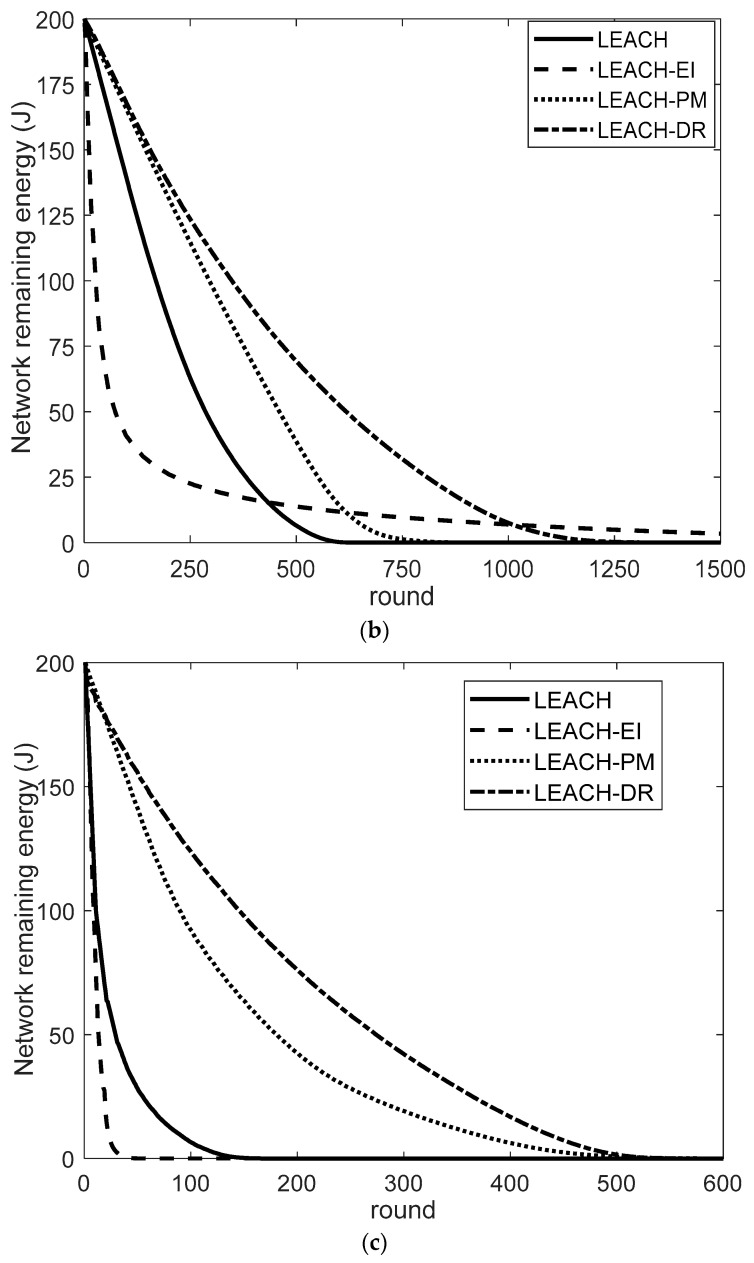
Comparison of network residual energy at different BS locations: (**a**) Comparison of network remaining energy at BS (0, 0); (**b**) Comparison of network remaining energy at BS (250, 250); (**c**) Comparison of network remaining energy at BS (250, 750).

The energy consumption directly affects the sustainable operation of the network, and the comparison of the remaining energy of the network of the four protocols with the operation of the network is shown in Figure 9a–c.

The LEACH-DR protocol shows significant performance advantages over LEACH, LEACH-PM, and LEACH-EI under different base station locations. As shown in Figure 9a, when the base station is at (0, 0), LEACH-DR significantly pushes the half-life of the network from 13 rounds for LEACH-EI, 30 rounds for LEACH, and 160 rounds for LEACH-PM to 232 rounds, and extends the total network lifetime from 630 rounds for LEACH and 843 rounds for LEACH-PM to 981 rounds, achieving a growth rate of 55.7% and 16.4% of the network lifetime. From the perspective of the overall residual energy curve of the network, the curve slope of LEACH-DR is always smaller than the other three; that is, the energy utilization rate is the highest, and the energy consumption rate is the most stable.

When the base station location is moved to the network center (250, 250), as shown in Figure 9b, the half-life of LEACH-DR appears in the 349th round, later than the 28th round for LEACH-EI, the 170th round for LEACH, and the 296th round for LEACH-PM. Its total survival period reached 1367 rounds, which also significantly exceeded the 654 rounds of LEACH and 917 rounds of LEACH-PM. The LEACH-DR protocol extends the network lifecycle by about 109% and 49.1%, respectively, compared to the two. From this curve, it can also be found that the final life cycle round of LEACH-DR is smaller than that of LEACH-EI, but on the whole, the energy decline curve of LEACH-DR is more gentle and stable, which indicates that its energy consumption rate is lower and the network load is more balanced, thus maintaining higher quality network coverage and data acquisition capabilities for most of the time.

As shown in Figure 9c, in the most challenging edge base station scenarios (250, 750), LEACH-DR demonstrates even more prominent performance advantages. Its network energy is exhausted at 578 rounds, far exceeding the 169 rounds of LEACH and 55 rounds of LEACH-EI. Although the remaining energy of LEACH-PM is exhausted in 622 rounds, the energy decline trend of LEACH-DR is significantly more gentle from the overall curve, indicating that it can still effectively coordinate node energy consumption under extreme network topologies, avoid local energy voids, and is more suitable for large-scale network coverage.

In summary, the LEACH-DR protocol can most effectively delay the energy exhaustion process of the network in the three base station location scenarios of the simulation experiment through an efficient CH election and routing mechanism, and the number of rounds to reach the critical energy exhaustion node is always significantly ahead of the LEACH-EI, LEACH-PM and LEACH protocols. Its ability to maintain network operation is the most prominent in the topology-unfavorable edge base station environment.

2. Comparison of node death rounds

The network stability is evaluated by the first node death time, as shown in Table 2. The four protocols show significant differences in node death time.

The experimental data show that the LEACH-DR protocol can significantly delay the process of network node failure in most scenarios. When the base station is at (0, 0), from the perspective of 25% node death rounds, LEACH-DR has a death round of 323, which is significantly better than the 32nd round of the LEACH protocol, the 251st round of the LEACH-PM protocol, and the 13th round of the LEACH-EI protocol. With the increase in node deaths, the advantages of the LEACH-DR protocol become more and more obvious, reaching 583 rounds when 50% of nodes die and 788 rounds when 75% of nodes die, all of which are significantly delayed compared to the other three protocols.

When the base station is located at the center of the network (250, 250), 25% of the LEACH-DR protocol’s node death rounds are delayed to 544 rounds, surpassing the 265 rounds of the LEACH protocol and the 32 rounds of the LEACH-EI protocol. Although it predates the 563 rounds of the LEACH-PM protocol, when 50% of nodes die, the LEACH-DR protocol reaches 902 rounds, ahead of the 69 rounds of the LEACH-EI protocol, the 380 rounds of the LEACH protocol, and the 624 rounds of the LEACH-PM protocol. When 75% of the nodes died, the LEACH-DR protocol reached 1045 rounds, which is significantly later than the other three protocols.

When the base station is located at the edge of the network (250, 750), 25% of the LEACH-DR protocol has 155 rounds of node deaths, surpassing the 12 rounds of the LEACH protocol, the 117 rounds of the LEACH-PM protocol, and the 8 rounds of the LEACH-EI protocol. This shows that LEACH-DR can still maintain the long-term stable operation of most networks through effective mechanisms after facing the initial node failure.

Overall, the LEACH-DR protocol has significant advantages in maintaining the overall lifetime of the network, especially in delaying large-scale node failures.

3. Comparison of the number of packets received by base stations

**Figure 10:**
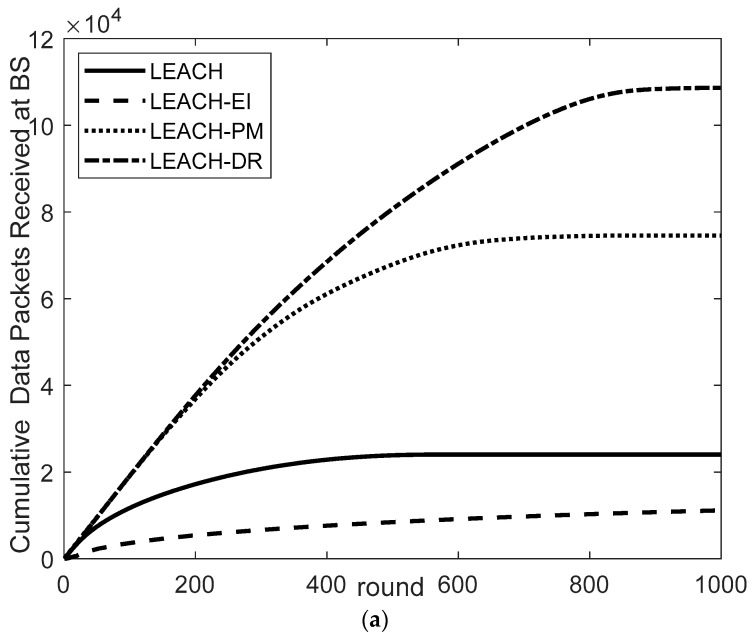

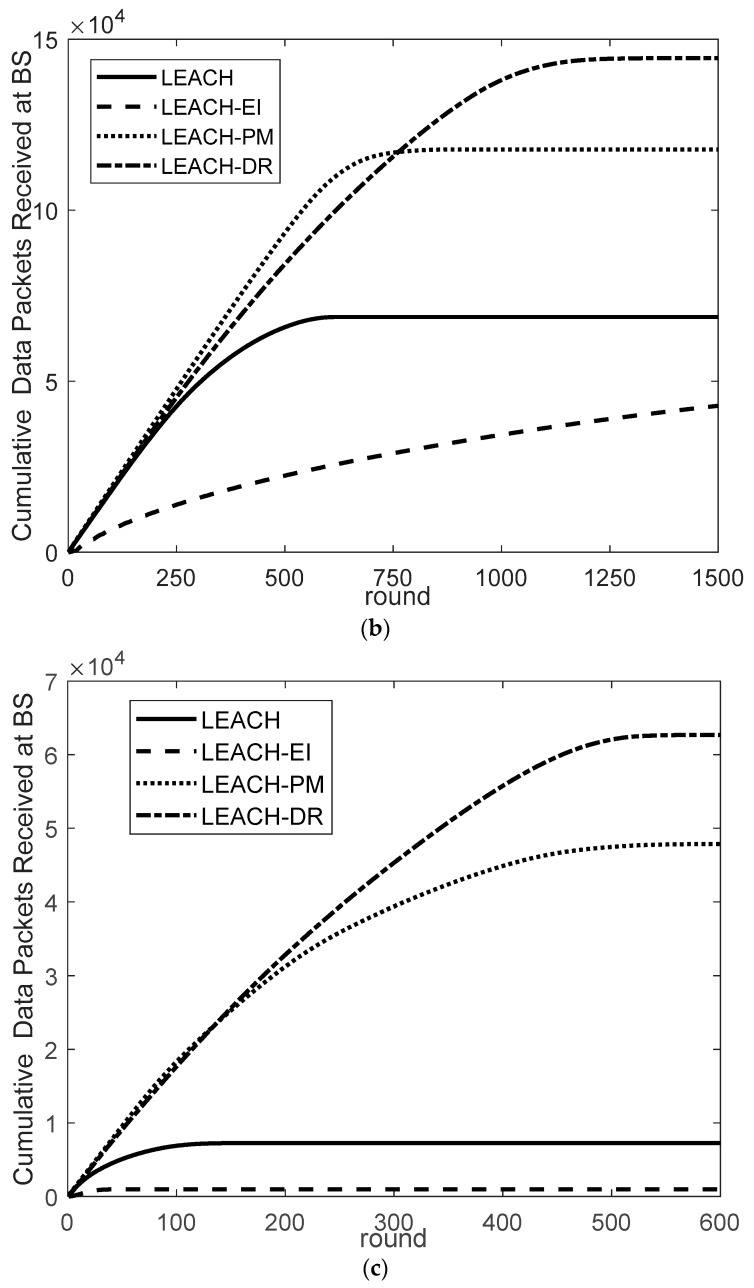
Comparison of the cumulative number of packets received by base stations at different locations: (**a**) Comparison of the cumulative number of packets received at the BS (0, 0); (**b**) Comparison of the cumulative number of packets received at the BS (250, 250); (**c**) Comparison of the cumulative number of packets received at the BS (250, 750).

From the experimental results in Figure 10a–c, it can be seen that the four protocols show significant differences in the cumulative data volume. In the performance evaluation of the number of packets received by the base station, the LEACH-DR protocol demonstrates excellent data throughput and network transmission efficiency. Through comparative testing of three typical base station locations, the significant advantages of this protocol in data collection can be clearly observed.

As shown in Figure 10a, when the base station is at the (0, 0) position, the LEACH-DR protocol receives a total of 10.86 × 104 packets, which is 1.46 times that of the LEACH-PM protocol (7.45 × 104), 4.52 times that of the LEACH protocol (2.4 × 104), and 8.6 times that of the LEACH-EI protocol (1.26 × 104). This result shows that LEACH-DR maintains efficient data transmission performance even when the base station position is offset from the center of the network.

As shown in Figure 10b, when the base station position is adjusted to (250, 250), the data collection capacity of LEACH-DR is further increased to 14.45 × 104, which is equivalent to 1.23 times that of the LEACH-PM protocol (11.78 × 104), 2.1 times that of the LEACH protocol (6.88 × 104), and 3.37 times that of the LEACH-EI protocol (4.28 × 104).

As shown in Figure 10c in the edge base station scenario with the most complex network topology (250, 750), the LEACH-DR protocol still maintains an excellent reception capacity of 6.27 × 104 packets, which is 1.31 times that of the LEACH-PM protocol (4.79 × 104), 8.64 times that of the LEACH protocol (7256), and 63.9 times that of the LEACH-EI protocol (981). This data highlights the LEACH-DR protocol’s strong adaptability in adverse network environments.

A series of data fully verifies the outstanding performance of the LEACH-DR protocol in network transmission efficiency, proves its remarkable results in the routing mechanism and data transmission optimization, and provides a reliable technical solution for data collection in large-scale network environments for WSN applications.

4. Energy efficiency comparison

**Figure 11:**
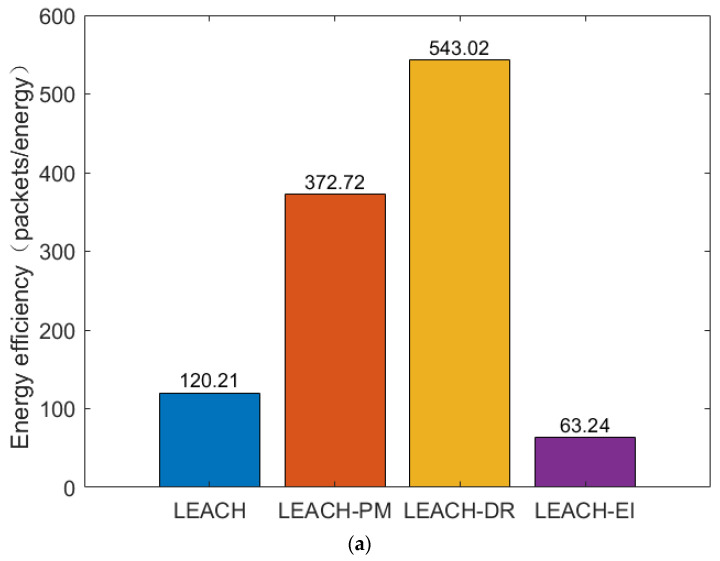

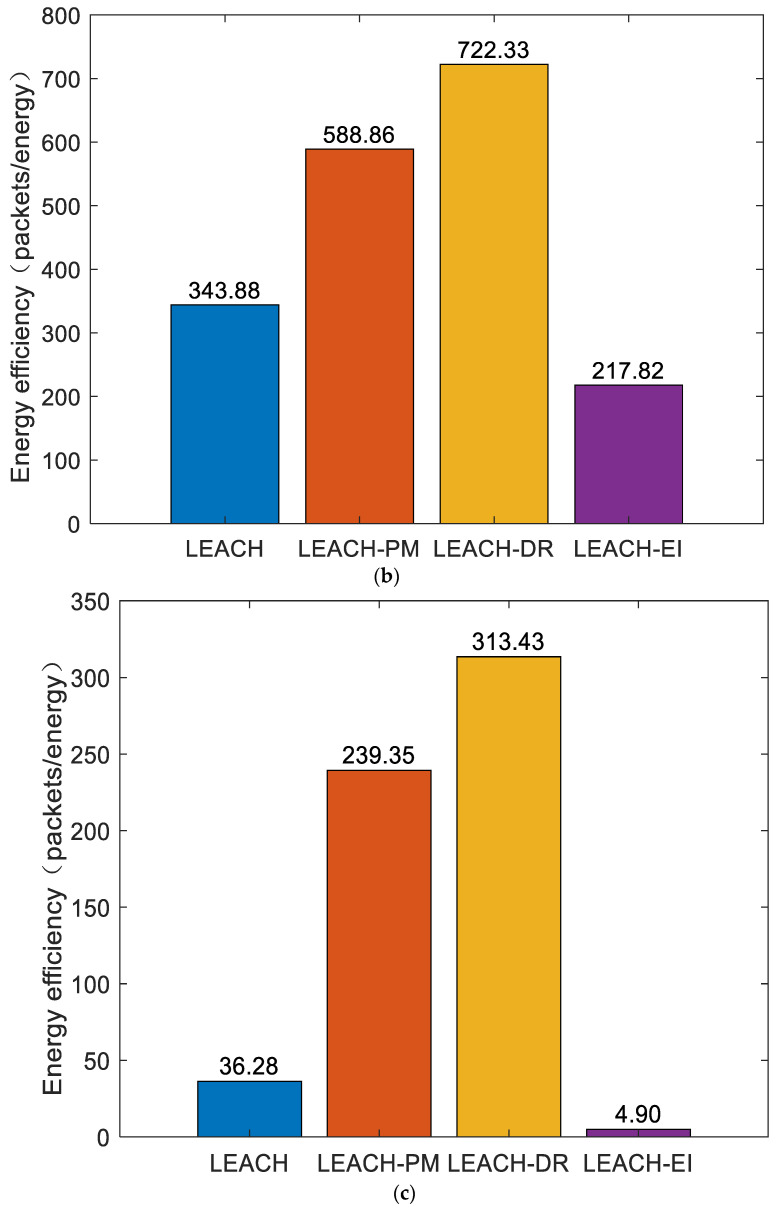
Comparison of energy efficiency at different base stations: (**a**) Comparison of energy efficiency at BS (0, 0); (**b**) Comparison of energy efficiency at BS (250, 250); (**c**) Comparison of energy efficiency at BS (250, 750).

Energy efficiency is an important indicator to measure the energy utilization efficiency of network protocols during data transmission. This study takes the ratio of the number of packets received by the base station to the total energy consumed by the network as the value of energy efficiency. The data in Figure 11a shows that the LEACH-DR protocol has the highest energy efficiency of 543.02 packets/joule when the base station is in the (0, 0) position, which is significantly better than LEACH-PM’s 372.72, LEACH’s 120.21, and LEACH-EI’s 63.24.

Figure 11b shows the energy efficiency of the four routing protocol base stations at (250, 250), with the LEACH-DR protocol leading with an efficiency of 722.33 packets/energy, indicating the best performance in energy management and data transmission. LEACH-PM is next with an efficiency of 588.86 packets/energy. The energy efficiencies of LEACH and LEACH-EI are relatively low at 343.88 and 217.82 packets/energy, respectively. These results highlight the potential of the LEACH-DR protocol to improve WSN energy efficiency.

Figure 11c reveals that the energy efficiency of the LEACH-DR protocol is still significantly better than that of the LEACH-PM and LEACH protocols when the base station is located at (250, 750), with energy efficiencies reaching 313.43, 239.35, and 36.28 packets/energy, respectively. The LEACH-EI protocol performed the worst, with only 4.90 packets/energy.

In summary, the LEACH-DR protocol demonstrates excellent performance in terms of energy efficiency, which has significant practical value for improving the overall energy efficiency of WSN and extending network life.

## 6. Conclusions

This paper proposes the LEACH-DR protocol based on dynamic clustering and routing optimization. It achieves dynamic clustering through a Self-Organizing Map (SOM) neural network, comprehensively considers residual energy, neighbor density, and distance to the BS during CH election, and employs a Fibonacci-inspired bees algorithm to plan efficient inter-cluster paths. The simulation results demonstrate that the proposed protocol significantly improves energy efficiency and network lifetime compared to LEACH, LEACH-PM, and LEACH-EI, proving particularly suitable for large-scale network deployment. However, this study still has the following limitations that provide directions for future research: the current model is based on the assumption of a static and homogeneous network, whereas in practice, node mobility (e.g., in mobile sensor networks) and heterogeneity (e.g., coexistence of energy-harvesting nodes and ordinary nodes) may affect clustering and routing stability; the simulation environment is idealized and does not fully account for real-world factors such as channel interference and link unreliability; and the protocol has not yet integrated security mechanisms, such as trust evaluation, defense against malicious nodes, and data encryption, which are crucial in practical WSN applications.

## Figures and Tables

**Table 1:** Network parameter settings.

Parameter	Value
Network range/m^2^	500 × 500
The number of nodes	200
Node initial energy/J	1
$\varepsilon_{fs}$/(pJ/bit/m^2^)	10
$\varepsilon_{mp}$/(pJ/bit/m^4^)	0.0013
$E_{elec}$/(nJ/bit)	50
$E_{DA}$/(nJ/bit)	5
packet size /bit	4000
Control information size/bit	32
α	0.4
β	0.3
γ	0.3
$\eta_{1}$	0.4
$\eta_{2}$	0.3
$\eta_{3}$	0.3
$R_{ci}$/m	20

**Table 2:** Comparison of node death rounds.

Protocol	BS Location	25%	50%	75%
LEACH	(0, 0)	32	68	191
	(250, 250)	265	380	512
	(250, 750)	12	26	64
LEACH-PM	(0, 0)	251	361	541
	(250, 250)	563	624	686
	(250, 750)	117	224	386
LEACH-EI	(0, 0)	13	21	43
	(250, 250)	32	69	233
	(250, 750)	8	13	20
LEACH-DR	(0, 0)	323	583	788
	(250, 250)	544	902	1045
	(250, 750)	155	371	471

## Data Availability

Data are contained within the article.
